# Novel Identification of Bacterial Epigenetic Regulations Would Benefit From a Better Exploitation of Methylomic Data

**DOI:** 10.3389/fmicb.2021.685670

**Published:** 2021-05-14

**Authors:** Amaury Payelleville, Julien Brillard

**Affiliations:** ^1^DGIMI, INRAE, Univ. Montpellier, Montpellier, France; ^2^Cellular and Molecular Microbiology, Faculté des Sciences, Université Libre de Bruxelles, Gosselies, Belgium

**Keywords:** bacterial epigenetics, DNA methylation, methylome, transcriptome, phenotypic heterogeneity

## Abstract

DNA methylation can be part of epigenetic mechanisms, leading to cellular subpopulations with heterogeneous phenotypes. While prokaryotic phenotypic heterogeneity is of critical importance for a successful infection by several major pathogens, the exact mechanisms involved in this phenomenon remain unknown in many cases. Powerful sequencing tools have been developed to allow the detection of the DNA methylated bases at the genome level, and they have recently been extensively applied on numerous bacterial species. Some of these tools are increasingly used for metagenomics analysis but only a limited amount of the available methylomic data is currently being exploited. Because newly developed tools now allow the detection of subpopulations differing in their genome methylation patterns, it is time to emphasize future strategies based on a more extensive use of methylomic data. This will ultimately help to discover new epigenetic gene regulations involved in bacterial phenotypic heterogeneity, including during host-pathogen interactions.

## Introduction

Epigenetic regulations have been studied mainly in eukaryotes where they are involved in cell differentiation or disease occurrence, through diverse mechanisms such as histone modifications or DNA methylation. However, evidences for the existence of epigenetic regulation in prokaryotes are increasingly reported (Sánchez-Romero and Casadesús, 2020). Such gene regulations can occur by feedback loops (positive or negative) but most examples involve DNA methylation (Adhikari and Curtis, 2016). DNA methylation is a base modification system that acts by the addition of a methyl group from an S-adenosyl-methionine molecule to an Adenine or a Cytosine in the DNA. In the growing cell, this process usually occurs shortly after the DNA replication on the newly synthesized strand. Enzymes responsible for DNA methylation are called DNA-methyltransferases (MTases) and catalyze three types of DNA methylation modifications: N^6^-methyl-adenine (m6A), C^5^-methyl-cytosine (m5C) and N^4^-methyl-cytosine (m4C). While all three types are described in archaea and bacteria, m4C modification is not reported in eukaryotes. Genes encoding putative MTases are found in almost all bacterial species, and most bacterial genomes analyzed so far display a DNA methylation pattern that is species or strain-specific (Blow et al., 2016). Many bacterial MTases belong to restriction-modification systems, as they are genetically and functionally associated to a restriction endonuclease (REase) that protects the bacterial cell from exogenous DNA (Marinus and Løbner-Olesen, 2014; Roberts et al., 2015). In addition, “solitary” or “orphan” MTases are frequently found in the genomes of bacteria (Blow et al., 2016) and some of them carry key roles in genome maintenance (Løbner-Olesen et al., 2005). DNA methylation can also affect the interaction of DNA-binding proteins with their cognate sites, either directly (e.g., steric hindrance) or by changes in DNA topology (Casadesús, 2016), resulting in epigenetic regulations (Casadesús and Low, 2013). This mechanism, among others, is responsible for prokaryotic phenotypic heterogeneity (Sánchez-Romero et al., 2020), a phenomenon of critical importance for successful infection by several major pathogens (Balaban et al., 2004; Weigel and Dersch, 2018).

Thanks to recent advances in long-read sequencing technologies, data of the methylated bases over the complete genome (methylome) of bacteria is progressively reported (Rand et al., 2017; Beaulaurier et al., 2019; García-Pastor et al., 2019). However, most of these technologies are exclusively used to generate new genomics data, while the methylomic data are often set aside. Thus, there is a gap between the scarce reports of bacterial gene regulation associated to DNA methylation and the increasing availability of unexploited methylomic data. Given the pervasiveness of DNA methylation in prokaryotes, we believe that a deeper analysis of methylomic data could lead to identifying new candidates of epigenetically regulated genes. This review illustrates the importance of DNA methylation associated to epigenetic regulations in bacteria and aims to raise awareness on the available yet underused tools, in the field of bacterial epigenetic.

## Bacterial Transcriptional Factors Involved in Epigenetic Regulation

**FIGURE 1:**
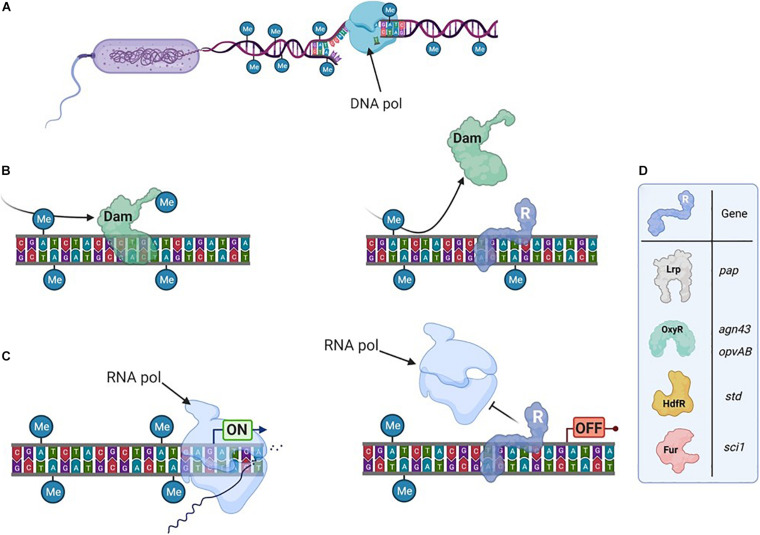
Transcription can depend on the DNA-methylation pattern. **(A)** The bacterial genome is usually broadly methylated. It is transiently hemimethylated after DNA-replication. **(B)** DNA-Methyltransferases methylate DNA on particular motifs. Here an Adenine in GATC motif is being methylated by Dam (left), unless a transcriptional regulator (R) hinders its access to the motif (right). After a second replication step, the DNA can become unmethylated. **(C)** Transcription is initiated by the RNA-polymerase (left) unless a transcriptional regulator is bound in the promoter region (right). **(D)** Examples (detailed in the text) of transcriptional regulators sensitive to DNA methylation.

While DNA methylation occurs on motifs located anywhere on the DNA, typical bacterial epigenetic regulations are located in promoter regions (Figure 1). The main examples are linked to m6A modifications and either involve (i) CcrM, an MTase found in several α-proteobacteria, where it plays a critical role in controlling the cell cycle (Mouammine and Collier, 2018); or (ii) Dam, an MTase conserved in Enterobacteriaceae, which is associated to the formation of subpopulations with distinct phenotypes (van der Woude and Baumler, 2004; Marinus and Løbner-Olesen, 2014; Adhikari and Curtis, 2016; Sánchez-Romero and Casadesús, 2020).

The affinity of some DNA-binding proteins, including transcriptional regulators, can be affected by the DNA methylation state within or in the vicinity of their binding sites. After DNA replication (Figure 1A), the future methylation state of the locus will depend on whether the regulator or the MTase binds the newly synthesized (unmethylated) DNA strand first (Figure 1B). Gene expression will subsequently differ based on if (or where) the regulator is bound in these promoter regions. Thereby, this mechanism can therefore give rise to two bacterial subpopulations with distinct transcription patterns and consequently distinct phenotypes (Figure 1C).

Examples of epigenetic regulations associated to Dam often involve genes playing critical roles during bacterial-host interaction. Several transcriptional regulators were shown to be sensitive to the methylation state of the promoter region they control (for a recent review, see Sánchez-Romero and Casadesús, 2020). A canonic example is the regulation of the pilus-encoding *pap* operon by Lrp, extensively reviewed elsewhere (van der Woude et al., 1992, 1996; Peterson and Reich, 2008). Nevertheless, other examples of epigenetic gene regulation involving important transcriptional regulators deserve to be mentioned here (Figure 1D). A competition between the OxyR transcriptional regulator and Dam has been described for several promoter regions, as *agn43* (encoding an outer membrane protein) in *E. coli* or *gtr* or *opvAB* (encoding proteins involved in LPS modification) in *Salmonella* (Waldron et al., 2002; Wallecha et al., 2002; Cota et al., 2016; Sánchez-Romero et al., 2020). Such epigenetic regulations direct the formation of subpopulations that have a distinct fitness depending on the environment. For instance in *Salmonella*, control of the *opvAB* operon by OxyR produces one subpopulation that is resistant to infection by many bacteriophages but avirulent, and another that is phage sensitive but able to infect animal hosts (Cota et al., 2015). HdfR is a transcriptional regulator involved in the epigenetic regulation of the *std* fimbrial operon (García-Pastor et al., 2019). Here, the regulation mechanism is complexified by a positive feedback loop involving two additional regulators (StdE and StdF) encoded by the *std* operon. The Fur transcriptional regulator is critical for the iron stress response (Escolar et al., 1999) and is also involved in the epigenetic regulation of the *sci1* operon, encoding a Type VI Secretion System (T6SS) in *E. coli* (Brunet et al., 2011; Brunet et al., 2020). In this case, an intraspecific bacterial competition occurs between the two subpopulations dependent on the active expression of the T6SS. In addition to these few selected examples, a list of other regulators sensitive to DNA methylation can be found elsewhere (Sánchez-Romero and Casadesús, 2020). Altogether, they illustrate that the DNA methylation pattern is important for several bacterial phenotypes.

## Bacterial Phenotypes Associated to Changes in DNA-Methylation Pattern

The DNA methylation pattern can be significantly impacted by environmental conditions, as observed in some α or γ-proteobacteria (Hale et al., 1994; Ichida et al., 2007; Doberenz et al., 2017). Yet, the methylome of several γ-proteobacteria appeared very stable despite various growth conditions tested (Cohen et al., 2016; Westphal et al., 2016; Payelleville et al., 2018). In many examples, major phenotypic modifications are driven by the modification of the DNA methylation pattern through mutation or overexpression of an MTase gene. For instance, *dam* mutation caused impaired virulence in *Salmonella*, *Klebsiella, Haemophilus*, *Yersinia*, and *Actinobacillus* (García-Del Portillo et al., 1999; Heithoff et al., 1999; Watson et al., 2004; Robinson et al., 2005; Taylor et al., 2005; Wu et al., 2006; Mehling et al., 2007). While *dam* deletion can sometimes be lethal for the bacterium (as in *V. cholerae* or *A. hydrophila)* (Julio et al., 2001; Erova et al., 2006) its overexpression often leads to a decreased virulence phenotype (Heithoff et al., 1999; Julio et al., 2002; Chen et al., 2003; Erova et al., 2012; Payelleville et al., 2017).

Other examples below illustrate the major phenotypes found in association with DNA-methylation pattern deregulation, whatever the type of methylated based involved. For most of these examples, the underlying mechanisms responsible for the modified phenotype has not been described yet. The CamA orphan m6A MTase conserved among all *C. difficile* is involved in sporulation and persistence and many genes in the MTase mutant are shown to be differentially regulated (Oliveira et al., 2020). Dcm, a broadly distributed m5C MTase in *Enterobacteriaceae*, plays a role during the stationary growth phase as its deletion leads to an increase in the RpoS sigma factor expression (Kahramanoglou et al., 2012; Militello et al., 2012, 2016, 2020) and it may also be linked to antimicrobial compound resistance (Militello et al., 2014). Furthermore, the deletion of m4C MTases encoding genes can impair virulence and cause broad transcriptional changes as shown in *H. pylori* (Kumar et al., 2012, 2018). In another human pathogen, *Leptospira interrogans*, the impaired virulence caused by the deletion of an MTase is associated to the dysregulation of an extracytoplasmic function (ECF) sigma factor (Gaultney et al., 2020).

The so-called “Phasevarion” (for phase-variable regulon) allows broad changes in DNA-methylation patterns. It occurs in bacteria that express MTases with phase variation character (i.e., a reversible switch in expression leading to single-cell phenotypic heterogeneity). This phenomenon provides a way to modify the expression of numerous genes simultaneously through epigenetic regulation by a single event of phase variation. This way, phasevarions confer the ability for the bacterium to adapt to broader environmental conditions. The phenomenon has been described in several major pathogens [*H. influenzae* (De Bolle et al., 2000), *H. pylori* (de Vries et al., 2002; Srikhanta et al., 2017b), *Streptococcus suis* (Atack et al., 2018b), S. *pneumoniae* (Manso et al., 2014), *Neisseria meningitidis* (Jen et al., 2014; Seib et al., 2015), *N. gonorrheae* (Srikhanta et al., 2009; Seib et al., 2017), *Moraxella catarrhalis* (Blakeway et al., 2014; Blakeway et al., 2019), *Kingella kingae* (Srikhanta et al., 2017a)] and is reviewed elsewhere (Atack et al., 2018a; Seib et al., 2020). Moreover, new bioinformatic tools allowing for the detection of genetic signatures of those events have been recently developed, giving hope for a further increase in the description of the underlying mechanism (Atack et al., 2020a,b). All the phasevarions described until now involve MTases that are part of RM systems. Given the significant proportion of type II solitary MTases in prokaryotes [more than 50%, (Blow et al., 2016)], and considering that they are involved in most described epigenetic regulations, we can hypothesize that phasevarion involving solitary MTases might be discovered in the future. Again, even though the expression switch of the MTase is well described, the exact epigenetic regulation mechanisms of the various regulon members remain to be elucidated. Modern technologies enabling the analysis of the cells’ methylome may help to answer these questions.

## Sequencing Methods to Analyze Bacterial Methylomes

Three main methods are currently used for the base resolution sequencing of DNA methylation (reviewed in Beaulaurier et al., 2019). They all require powerful bioinformatic tools to have an accurate and complete view of the modified bases in the whole genome.

(I) The Whole Genome Bisulfite Sequencing (WGBS) has been used for years, mostly in eukaryotes. In this approach, unmethylated cytosines are converted into uracils by the bisulfite treatment. After sequencing (usually short-read Illumina sequencing) and alignment, m5C can then be identified (Carless, 2009). This tool which is specific to the detection of m5C has found scarce application for the bacterial methylome analysis so far (Kahramanoglou et al., 2012). (II) The possibility to use the long-range method SMRT sequencing (for Single Molecule Real Time) to detect DNA methylation at a genomic scale was described in 2010 (Flusberg et al., 2010; Cloney, 2016). This technology makes use of a DNA polymerase sensitive to base modifications. Upon recognition of a modified base on the ssDNA matrix, a delay in polymerization is generated during the recording of the DNA sequence (InterPulse Duration or IPD). While it can easily detect m4C and m6A, this technology has strong limitations to detect m5C (requires a substantial coverage rate). Therefore, performing both WGBS and SMRT sequencing can allow to determine an exhaustive methylome, something rarely done (Payelleville et al., 2018; Vandenbussche et al., 2021). (III) More recently, another long-range sequencing method, the Oxford Nanopore technology (ONT), was shown to efficiently detect modified bases. While ssDNA crosses nanopores embedded in a lipid membrane, a voltage potential is applied. Analysis of the electrolytic current signals, which are sensitive to base modifications, reveals both the DNA sequence and the methylation state of the DNA matrix (Rand et al., 2017; Simpson et al., 2017). The earliest studies using ONT were focused on m5C detection of CpG islands, found in some eukaryotes (Laszlo et al., 2013), but various bioinformatic models have been developed since to increase the accuracy of other DNA methylation predictions. In certain DNA motifs, such models are now able to detect m5C and m6A using a low read coverage (as low as twofold) (Ni et al., 2019) with a significant precision on *E. coli* data (Liu et al., 2019). Recently, the methylomes of eight microbial reference species have been validated using various methods, including ONT sequencing for m6A detection (McIntyre et al., 2019).

The increasing number of bacterial metagenomic studies which use such sequencing technologies (Petersen et al., 2019; Tourancheau et al., 2021) is about to expand the set of data that could be used in parallel for analyzing DNA methylation. This, in turn, could stimulate bacterial epigenetic research. In addition, because the DNA methylation pattern is often strain-dependent, incorporation of methylomic information into shotgun metagenomic analyses was proposed as a new tool for distinguishing genomes of closely related strains, hence providing a much more accurate clustering of metagenomic sequences (Beaulaurier et al., 2018). If such strategy became widespread in the future, it would reinforce the importance of exploiting methylomic data.

## Combining Methylome Analysis With Other Approaches to Identify Putative Epigenetic Regulation

Numerous examples, as illustrated above, have demonstrated that the DNA methylation pattern at a given site can impact gene expression (Sánchez-Romero and Casadesús, 2020). Therefore, in a bacterial genome, each subset of unmethylated recognition motifs could be considered as a putative epigenetic regulatory site (Figure 1). Such approach was recently proved to be strikingly efficient in *Salmonella* and led to the identification of several new genes displaying expression heterogeneity controlled by Dam-methylation (Sánchez-Romero et al., 2020). A broad conservation among prokaryotes of unmethylated sites that are usually recognized by conserved MTases (Dam in γ-proteobacteria, or CcrM in α-proteobacteria) (Blow et al., 2016) strengthens the hypothesis of a widespread occurrence of this regulatory mechanism in bacteria.

**FIGURE 2:**
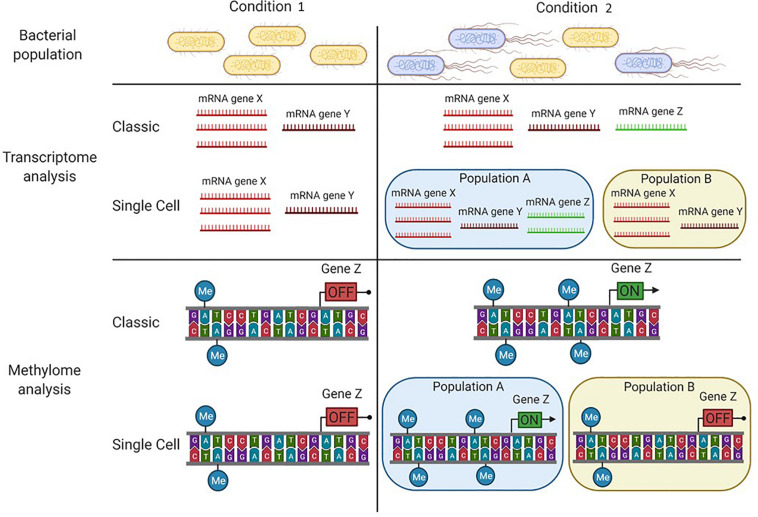
Particular conditions can lead to bacterial subpopulations in an isogenic population. In condition 1, the bacterial population has an homogeneous phenotype where individual cells display a similar transcription pattern and the same DNA-methylation pattern. In condition 2, two subpopulations are present (**A** and **B**), each one displaying a particular transcription pattern and a particular DNA-methylation pattern. While classical tools (e.g., SMRT sequencing and RNA seq analysis) allow the detection of differences between each condition, only the major subpopulation **(A)** is considered. To distinguish the two subpopulations, single cell tools need to be applied (e.g., SMALR for DNA methylation and Record-seq or PETRI-seq for transcription).

Given the large amount of transcriptomic data released in the databases, together with the increasing acquisition of genomic data by the help of technologies that also allow to identify the DNA methylation pattern (SMRT or ONT), we propose the coupling of transcriptome analysis with extensive methylome analysis. It may lead to the identification of putative epigenetic regulation networks. This would be particularly true if unmethylated motifs located in gene regulatory regions (i.e., promoters) correlate with differential regulation of the genes in an altered environmental condition (or in a mutant strain, Figure 2).

Up to now, this combined methylome/transcriptome strategy has been rarely employed and led to the identification of only a limited number of candidate genes under a putative epigenetic regulation, which still need to be confirmed by additional mechanistic studies (Blow et al., 2016; Cohen et al., 2016; Doberenz et al., 2017; Payelleville et al., 2018; Nye et al., 2019). This low occurrence of candidates has to be balanced by the fact that a complex gene regulation may require multiple factors for a fine tuning of expression, as exemplified by the epigenetic regulation of the Std fimbriae in *Salmonella* described above (García-Pastor et al., 2019). Furthermore, the contribution of nucleoid associated proteins, such as H-NS, in epigenetic mechanisms involving DNA-methylation has also been reported in various cases and therefore also contribute to the formation of bacterial subpopulations (Nicholson and Low, 2000; Camacho et al., 2005; Cota et al., 2016).

## Bacterial Phenotypic Heterogeneity: Methylome and Transcriptome Analyses of Subpopulations

The methylome analysis methods described above determine the DNA methylation to the nucleotide resolution at the population level. However, distinct DNA methylation patterns can drive the emergence of different subpopulations with different expression profiles (Figure 2). In 2015, an improvement in SMRT sequencing, SMALR for Single Molecule modification Analysis of Long Reads, was proposed (Beaulaurier et al., 2015). The enhancement resides on the ability of SMALR to identify epigenetic heterogeneity, where a subpopulation displays a distinct methylation pattern compared to the rest of the population. Despite its potential to identify subpopulations with different methylation patterns, currently few studies reporting the use of SMALR can be found in the literature (Modlin et al., 2020). This may be due to an ongoing need for improved or analysis-specific tools based on modern sequencing technologies to decipher the mechanisms that give rise to the formation of subpopulations in an isogenic bacterial culture.

Importantly, two tools were recently designed to study complete gene expression at the subpopulation level: (i) Record-seq (Schmidt et al., 2018) can report a change in gene expression during a bottleneck situation where the amount of events is too low for a classic global transcriptomic analysis (Schmidt et al., 2018; Tanna et al., 2020); (ii) PETRI-seq (Blattman et al., 2020) allows to detect subpopulations with a different transcriptomic profile where less mRNA is needed, compared to classic transcriptional studies. Combining the SMALR and one of those transcriptomic tools should drastically improve the detection of candidate genes subject to DNA methylation regulation and heterogeneously expressed among a population.

In parallel, as prokaryotic phenotypic heterogeneity is of critical importance for a successful infection by various major pathogens, it is crucial that, in the near future, evolution of methylome analysis (supported by both improved sequencing coverage rates, and development of appropriate computational tools) will be more sensitive to allow for the distinction of differential DNA methylation patterns among a single DNA sample. This will increase the possibility to identify heterogeneity in epigenetic marks between bacterial subpopulations, including *in vivo* during bacterial infections.

## Conclusion

It is now time to consider the large amount of available data that could be thoroughly exploited in order to identify new candidates of putative epigenetic regulation. Although it might often be challenging to confirm such mechanisms of regulation, the candidates detected would most likely unveil major roles in the life cycle of the bacteria. This assumption is after all exemplified by the mechanisms of epigenetic regulation which have been deciphered up to now (van der Woude et al., 1996; Wallecha et al., 2002; Camacho et al., 2005; van der Woude and Henderson, 2008; Brunet et al., 2011; Cota et al., 2016; García-Pastor et al., 2019).

## Author Contributions

AP and JB designed and wrote the manuscript and designed the figures using Biorender.com. Both the authors contributed to the article and approved the submitted version.

## Conflict of Interest

The authors declare that the research was conducted in the absence of any commercial or financial relationships that could be construed as a potential conflict of interest.
